# Supplementary material for: Female top managers and firm performance
Source: PLoS One. 2023 Feb 15;18(2):e0273976. doi: 10.1371/journal.pone.0273976 (PMC9931137; doi:10.1371/journal.pone.0273976)
Supplement: S1 Table — (DOCX) [file pone.0273976.s001.docx]

**S1 Table. List of countries and years surveyed by world region**

| **SSA=Sub-Saharan Africa** | **obs** | **SSA (Cont.)** | **obs** | **Non-OECD-HI** | **obs** |
| --- | --- | --- | --- | --- | --- |
| Angola2006 | 425 | Rwanda2011 | 241 | Antiguaandbarbuda2010 | 151 |
| Angola2010 | 360 | Senegal2007 | 506 | Bahamas2010 | 150 |
| Benin2009 | 150 | Senegal2014 | 601 | Barbados2010 | 150 |
| Benin2016 | 150 | Sierra Leone2009 | 150 | Croatia2007 | 633 |
| Botswana2006 | 342 | SouthAfrica2007 | 937 | Croatia2013 | 360 |
| Botswana2010 | 268 | Southsudan2014 | 738 | Latvia2009 | 271 |
| BurkinaFaso2009 | 394 | Sudan2014 | 662 | Latvia2013 | 336 |
| Burundi2006 | 270 | Swaziland2006 | 307 | Lithuania2009 | 276 |
| Burundi2014 | 157 | Tanzania2006 | 419 | Lithuania2013 | 270 |
| Cameroon2009 | 363 | Tanzania2013 | 813 | Russia2009 | 1,004 |
| CapeVerde2009 | 156 | Togo2009 | 155 | Russia2012 | 4,220 |
| Centralafricanrepublic2011 | 150 | Uganda2006 | 563 | StKittsandNevis2010 | 150 |
| Chad2009 | 150 | Uganda2013 | 762 | TrinidadandTobago2010 | 370 |
| Congo2009 | 151 | Zambia2007 | 484 | Uruguay2006 | 621 |
| Côte d’Ivoire2009 | 526 | Zambia2013 | 720 | Uruguay2010 | 607 |
| DRC2006 | 340 | Zimbabwe2011 | 599 | Total | 9,569 |
| DRC2010 | 359 | Total | 29,008 | **OECD-HI** |  |
| DRC2013 | 529 | **EAP=East Asia and Pacific** |  | Chile2006 | 1,017 |
| Eritrea2009 | 179 | Cambodia2013 | 472 | Chile2010 | 1,033 |
| Ethiopia2011 | 644 | Cambodia2016 | 373 | Czech Republic2009 | 250 |
| Ethiopia2015 | 848 | China2012 | 2,700 | Czech Republic2013 | 254 |
| Gabon2009 | 179 | Fiji2009 | 164 | Estonia2009 | 273 |
| Gambia2006 | 174 | Indonesia2009 | 1,444 | Estonia2013 | 273 |
| Ghana2007 | 494 | Indonesia2015 | 1,320 | Israel2013 | 483 |
| Ghana2013 | 720 | LaoPDR2009 | 360 | Poland2009 | 455 |
| Guinea2006 | 223 | LaoPDR2012 | 270 | Poland2013 | 542 |
| GuineaBissau2006 | 159 | LaoPDR2016 | 368 | Slovak Republic2009 | 275 |
| Kenya2007 | 657 | Malaysia2015 | 1,000 | Slovak Republic2013 | 268 |
| Kenya2013 | 781 | Micronesia2009 | 68 | Slovenia2009 | 276 |
| Lesotho2009 | 151 | Mongolia2009 | 362 | Slovenia2013 | 270 |
| Lesotho2016 | 150 | Mongolia2013 | 360 | Sweden2014 | 600 |
| Liberia2009 | 150 | Myanmar2014 | 632 | Total | 6,269 |
| Madagascar2009 | 445 | PapuaNewGuinea2015 | 65 |  |  |
| Madagascar2013 | 532 | Philippines2009 | 1,326 |  |  |
| Malawi2009 | 150 | Philippines2015 | 1,335 |  |  |
| Malawi2014 | 523 | Samoa2009 | 109 |  |  |
| Mali2007 | 490 | Solomon Islands2015 | 151 |  |  |
| Mali2010 | 360 | Thailand2016 | 1,000 |  |  |
| Mauritania2006 | 237 | Timor Leste2009 | 150 |  |  |
| Mauritania2014 | 150 | Timor-Leste2015 | 126 |  |  |
| Mauritius2009 | 398 | Tonga2009 | 150 |  |  |
| Mozambique2007 | 479 | Vanuatu2009 | 128 |  |  |
| Namibia2006 | 329 | Vietnam2009 | 1,053 |  |  |
| Namibia2014 | 580 | Vietnam2015 | 996 |  |  |
| Niger2009 | 150 | Total | 16,482 |  |  |
| Nigeria2007 | 1,891 |  |  |  |  |
| Nigeria2014 | 2,676 |  |  |  |  |
| Rwanda2006 | 212 |  |  |  |  |
| **SAR=South Asia Region** | **obs** | **ECA (Cont.)** | **obs** | **LAC (Cont.)** | **obs** |
| Afghanistan2008 | 535 | Serbia2009 | 388 | StLucia2010 | 150 |
| Afghanistan2014 | 410 | Serbia2013 | 360 | StVincentandGrenadines2010 | 154 |
| Bangladesh2007 | 1,504 | Tajikistan2008 | 360 | Suriname2010 | 152 |
| Bangladesh2013 | 1,442 | Tajikistan2013 | 359 | Venezuela2006 | 500 |
| Bhutan2009 | 250 | Turkey2008 | 1,152 | Venezuela2010 | 320 |
| Bhutan2015 | 253 | Turkey2013 | 1,344 | Total | 22,057 |
| India2014 | 9,281 | Ukraine2008 | 851 | **MENA=Middle East and North Africa** |  |
| Nepal2009 | 368 | Ukraine2013 | 1,002 | Djibouti2013 | 266 |
| Nepal2013 | 482 | Uzbekistan2008 | 366 | Egypt2013 | 2,897 |
| Pakistan2007 | 935 | Uzbekistan2013 | 390 | Iraq2011 | 756 |
| Pakistan2013 | 1,247 | Total | 17,941 | Jordan2013 | 573 |
| SriLanka2011 | 610 | **LAC= Latin America and Caribbean** |  | Lebanon2013 | 561 |
| Total | 17,317 | Argentina2006 | 1,063 | Morocco2013 | 407 |
| **ECA=Eastern Europe and Central Asia** |  | Argentina2010 | 1,054 | Tunisia2013 | 592 |
| Albania2007 | 304 | Belize2010 | 150 | West Bank And Gaza2013 | 434 |
| Albania2013 | 360 | Bolivia2006 | 613 | Yemen2010 | 477 |
| Armenia2009 | 374 | Bolivia2010 | 362 | Yemen2013 | 353 |
| Armenia2013 | 360 | Brazil2009 | 1,802 | Total | 7,316 |
| Azerbaijan2009 | 380 | Colombia2006 | 1,000 |  |  |
| Azerbaijan2013 | 390 | Colombia2010 | 942 |  |  |
| Belarus2008 | 273 | Costarica2010 | 538 |  |  |
| Belarus2013 | 360 | Dominica2010 | 150 |  |  |
| Bosnia and Herzegovina2009 | 361 | Dom.Rep.2010 | 360 |  |  |
| Bosnia and Herzegovina2013 | 360 | Ecuador2006 | 658 |  |  |
| Bulgaria2007 | 1,015 | Ecuador2010 | 366 |  |  |
| Bulgaria2009 | 288 | ElSalvador2006 | 693 |  |  |
| Bulgaria2013 | 293 | ElSalvador2016 | 719 |  |  |
| Fyr Macedonia2009 | 366 | Elsalvador2010 | 360 |  |  |
| Fyr Macedonia2013 | 360 | Grenada2010 | 153 |  |  |
| Georgia2008 | 373 | Guatemala2006 | 522 |  |  |
| Georgia2013 | 360 | Guatemala2010 | 590 |  |  |
| Hungary2009 | 291 | Guyana2010 | 165 |  |  |
| Hungary2013 | 310 | Honduras2006 | 436 |  |  |
| Kazakhstan2009 | 544 | Honduras2010 | 360 |  |  |
| Kazakhstan2013 | 600 | Jamaica2010 | 376 |  |  |
| Kosovo2009 | 270 | Mexico2006 | 1,480 |  |  |
| Kosovo2013 | 202 | Mexico2010 | 1,480 |  |  |
| Kyrgyz Republic2009 | 235 | Nicaragua2006 | 478 |  |  |
| Kyrgyz Republic2013 | 270 | Nicaragua2010 | 336 |  |  |
| Moldova2009 | 363 | Panama2006 | 604 |  |  |
| Moldova2013 | 360 | Panama2010 | 365 |  |  |
| Montenegro2009 | 116 | Paraguay2006 | 613 |  |  |
| Montenegro2013 | 150 | Paraguay2010 | 361 |  |  |
| Romania2009 | 541 | Peru2006 | 632 |  |  |
| Romania2013 | 540 | Peru2010 | 1,000 |  |  |

Source: World Bank Enterprise Surveys, 2016.
