# Supplementary material for: Female top managers and firm performance
Source: PLoS One. 2023 Feb 15;18(2):e0273976. doi: 10.1371/journal.pone.0273976 (PMC9931137; doi:10.1371/journal.pone.0273976)
Supplement: S2 Table — (DOCX) [file pone.0273976.s002.docx]

**S2 Table. Number of firms surveyed by year and region**

|  | \|  \|  \|  \|  \| **Region** \|  \|  \|  \|  \|  \| \| --- \| --- \| --- \| --- \| --- \| --- \| --- \| --- \| --- \| --- \| \| **year** \| **SSA** \| **EAP** \| **ECA** \| **LAC** \| **MENA** \| **SAR** \| **OECD-HI** \| **Non-OECD-HI** \| **Total** \| \| 2008 \| 2,010 \| 215 \| 7,490 \| 0 \| 0 \| 535 \| 957 \| 1,071 \| 12,278 \| \| 2009 \| 1,987 \| 4,917 \| 402 \| 0 \| 0 \| 617 \| 572 \| 480 \| 8,975 \| \| 2010 \| 1,347 \| 180 \| 0 \| 5,921 \| 477 \| 0 \| 941 \| 602 \| 9,468 \| \| 2011 \| 1,374 \| 0 \| 0 \| 4,323 \| 756 \| 610 \| 92 \| 3,868 \| 11,023 \| \| 2012 \| 328 \| 778 \| 464 \| 0 \| 0 \| 0 \| 0 \| 1,326 \| 2,896 \| \| 2013 \| 3,501 \| 323 \| 7,568 \| 0 \| 2,776 \| 5,784 \| 1,808 \| 966 \| 22,726 \| \| 2014 \| 6,461 \| 894 \| 698 \| 0 \| 3,307 \| 6,737 \| 882 \| 0 \| 18,979 \| \| 2015 \| 1,647 \| 3,707 \| 0 \| 0 \| 0 \| 594 \| 0 \| 0 \| 5,948 \| \| 2016 \| 361 \| 2,765 \| 0 \| 719 \| 0 \| 0 \| 0 \| 0 \| 3,845 \| \| Total \| 19,016 \| 13,779 \| 16,622 \| 10,963 \| 7,316 \| 14,877 \| 5,252 \| 8,313 \| 96,138 \| |  |  |
| --- | --- | --- | --- | --- | --- | --- | --- | --- | --- | --- | --- | --- | --- | --- | --- | --- | --- | --- | --- | --- | --- | --- | --- | --- | --- | --- | --- | --- | --- | --- | --- | --- | --- | --- | --- | --- | --- | --- | --- | --- | --- | --- | --- | --- | --- | --- | --- | --- | --- | --- | --- | --- | --- | --- | --- | --- | --- | --- | --- | --- | --- | --- | --- | --- | --- | --- | --- | --- | --- | --- | --- | --- | --- | --- | --- | --- | --- | --- | --- | --- | --- | --- | --- | --- | --- | --- | --- | --- | --- | --- | --- | --- | --- | --- | --- | --- | --- | --- | --- | --- | --- | --- | --- | --- | --- | --- | --- | --- | --- | --- | --- | --- | --- | --- | --- | --- | --- | --- | --- | --- | --- | --- | --- |

**Source:** World Bank Enterprise Surveys, 2016. SSA= Sub-Saharan Africa; EAP=East Asia and Pacific; ECA=Eastern Europe and Central Asia; LAC= Latin America and the Caribbean; MENA=Middle East and North Africa; SAR= South Asia Region; HI OECD= High Income Organization for Economic Cooperation and Development; HI NOECD= High Income no members of the Organization for Economic Cooperation and Development.
