# Supplementary material for: Female top managers and firm performance
Source: PLoS One. 2023 Feb 15;18(2):e0273976. doi: 10.1371/journal.pone.0273976 (PMC9931137; doi:10.1371/journal.pone.0273976)
Supplement: S3 Table — (DOCX) [file pone.0273976.s003.docx]

**S3 Table. Variables definitions**

| **Category** | **Variable Acronym** | **Variable Definition** | **Question or Description** | **Question Code** |
| --- | --- | --- | --- | --- |
| **Gender** | **fem** | Dummy variable that takes the value of 1 if there is at least 1 female among the owners | Amongst the owners of the firm, are there any females? | b4 |
|  | **tfem** | Dummy variable that takes the value of 1 if the top manager is a female | Is the top manager female? | b7a |
|  | **femmore** | Dummy variable that takes the value if 1 if fem_cat>2 (at least 50% are female owners) | Are the owner of the firm: 1:all men, 2:mayority men,3:mayority women,4:all women,5:equaly divided | b4a_cat and author’s elaboration |
|  | **femopc** | Percentage of the firm owned by females | What percentage of the firm is owned by females? | b4a |
|  | **femempl** | Number of female employees | Number of employees who were female at end of last fiscal year | l5 |
| **Total Factor productivity (TPF)** | **capital** | Net book value of machinery vehicles, and equipment in last fiscal year | Net book value of machinery vehicles, and equipment in last fiscal year | na6 and author’s  elaboration |
|  | **materials** | Total purchases of raw material and intermediate goods (deflated by the production price index (PPI) for manufacturers) | Cost of raw materials and intermediate goods used in prod. in the last fiscal year | n6a and author’s  elaboration |
|  | **wages** | total labor cost (incl. wages, salaries, bonuses, etc.) in the last fiscal year (deflated by the production price index (PPI) for manufacturers) | Total cost of labor, including wages, salaries, and bonuses | n2a author’s  elaboration |
| **Ownership** | **foreign** | Dummy variable that takes the value of 1 if the firm is partly owned by a foreigner | Percentage of the firm owned by a foreign owner | b2b and author’s elaboration |
|  | **ownconc** | Percentage of the firm owned by the main owner | What percentage of this firm does the largest owner(s) own? | b3 |
| **International**  **Trade** | **exporter** | Dummy variable that takes value 1 if firm exports in year t | What percent of your establishment’s sales were exported directly in current year | Author’s elaboration from variables d3b and d3c (direct and indirect export shares) |

| **Investment Climate Constraints (Business environment)** | **accessfinance** | Access to Financing (Ex: Collateral) | Please tell us if any of the following issues are a problem for the operation and growth of your business. If an issue poses a problem, please judge its severity as an obstacle on a four-point scale where:  1 denotes major  2 denotes moderate  3 denotes minor  4 denotes no obstacle | k30 |
| --- | --- | --- | --- | --- |
|  | **corruption** | Corruption |  | j30f |
|  | **informal** | Illegal Competition from the informal sector/smuggling and dumping |  | e30 |
|  | **crime theft, disorder, and crimes** | theft, disorder, and crimes |  | i30 |
| **Education, Experience** | **exper** | Years of experience of the top manager | How many years of experience working in this sector does the top manager have? | b7 |
|  | **edufem** | Average years of education females | Average number of years of education of typical female | l9a2 |

Source: World Bank Enterprise Surveys, 2016.
