# Supplementary material for: Female top managers and firm performance
Source: PLoS One. 2023 Feb 15;18(2):e0273976. doi: 10.1371/journal.pone.0273976 (PMC9931137; doi:10.1371/journal.pone.0273976)
Supplement: S4 Table — (DOCX) [file pone.0273976.s004.docx]

**S4 Table. Summary statistics**

| **Variable** | **Obs** | **Mean** | **Std. Dev.** | **Min** | **Max** |
| --- | --- | --- | --- | --- | --- |
| **fem** | 60,937 | 0.308 | 0.462 | 0 | 1 |
| **tfem** | 60,937 | 0.154 | 0.361 | 0 | 1 |
| **femmore** | 35,705 | 0.133 | 0.340 | 0 | 1 |
| **femempl** | 26,929 | 26.117 | 305.284 | 0 | 38400 |
| **labp** | 60,937 | 98.029 | 471.274 | 0 | 64000 |
| **TFP** | 21,887 | 0.014 | 0.856 | -12.765 | 13.128 |
| **femopc** | 9,182 | 51.228 | 36.524 | 0 | 100 |
| **lnsales** | 60,937 | 16.956 | 3.248 | 0 | 33.846 |
| **lnlc** | 56,328 | 14.949 | 3.144 | 0 | 30.575 |
| **lnmat** | 30,023 | 16.160 | 3.489 | 0 | 32.013 |
| **lnk** | 23,123 | 15.555 | 3.343 | 0 | 32.813 |
| **lnvapw** | 33,668 | 13.164 | 2.809 | -1.897 | 27.252 |
| **lnlabpro** | 60,937 | 13.600 | 2.830 | -3.401 | 28.173 |
| **age** | 60,937 | 18.284 | 15.093 | 0.5 | 203 |
| **exper** | 60,937 | 17.062 | 10.689 | 1 | 59 |
| **crime** | 60,937 | 1.088 | 1.245 | 0 | 4 |
| **informal** | 60,937 | 1.432 | 1.345 | 0 | 4 |
| **corruption** | 60,937 | 1.694 | 1.453 | 0 | 4 |
| **accesfinance** | 60,937 | 1.460 | 1.305 | 0 | 4 |
| **owncon** | 60,937 | 0.786 | 0.266 | 0.01 | 1 |
| **exporter** | 60,937 | 0.231 | 0.422 | 0 | 1 |
| **foreign** | 60,937 | 0.064 | 0.224 | 0 | 1 |

Note: See Table S3 for variables definition.
