# Supplementary material for: Female top managers and firm performance
Source: PLoS One. 2023 Feb 15;18(2):e0273976. doi: 10.1371/journal.pone.0273976 (PMC9931137; doi:10.1371/journal.pone.0273976)
Supplement: S5 Table — (DOCX) [file pone.0273976.s005.docx]

**S5 Table. Pairwise correlations**

|  | **fem** | **tfem** | **lnsales** | **lnvapw** | **lnlabpro** | **age** | **experience** | **crime** | **informal** | **corruption** | **accessfinance** | **owncon** | **exporter** | **foreign** |
| --- | --- | --- | --- | --- | --- | --- | --- | --- | --- | --- | --- | --- | --- | --- |
| **fem** | 1 |  |  |  |  |  |  |  |  |  |  |  |  |  |
| **tfem** | 0.4148* | 1 |  |  |  |  |  |  |  |  |  |  |  |  |
| **lnsales** | 0.0371* | -0.0025 | 1 |  |  |  |  |  |  |  |  |  |  |  |
| **lnvapw** | 0.0098 | 0.0225* | 0.8752* | 1 |  |  |  |  |  |  |  |  |  |  |
| **lnlabpro** | 0.0160* | 0.0301* | 0.9029* | 0.9833* | 1 |  |  |  |  |  |  |  |  |  |
| **age** | 0.0529* | -0.0361* | 0.1119* | -0.0199* | 0.0026 | 1 |  |  |  |  |  |  |  |  |
| **exper** | 0.0518* | -0.0573* | 0.0241* | -0.0634* | -0.0441* | 0.3879* | 1 |  |  |  |  |  |  |  |
| **crime** | -0.0103 | -0.0061 | -0.0593* | -0.0709* | -0.0520* | 0.0190* | 0.0378* | 1 |  |  |  |  |  |  |
| **informal** | 0.0154* | -0.0047 | -0.0555* | -0.0296* | -0.0179* | 0.0308* | 0.0557* | 0.3094* | 1 |  |  |  |  |  |
| **corruption** | -0.0555* | -0.0593* | -0.0697* | -0.1021* | -0.0835* | 0.0343* | 0.0636* | 0.3951* | 0.2763* | 1 |  |  |  |  |
| **accessfinance** | -0.0184* | -0.0176* | -0.0912* | -0.0464* | -0.0406* | -0.0348* | -0.0247* | 0.2816* | 0.2791* | 0.2539* | 1 |  |  |  |
| **owncon** | -0.1802* | 0.0098 | -0.1233* | 0.0177* | -0.0038 | -0.1564* | -0.1339* | -0.0156* | 0.005 | -0.0528* | 0.0345* | 1 |  |  |
| **exporter** | 0.0571* | -0.0081 | 0.1634* | 0.0147* | 0.0077 | 0.1260* | 0.1114* | -0.0163* | -0.0403* | 0.0270* | -0.0402* | -0.1558* | 1 |  |
| **foreign** | -0.0261* | -0.0221* | 0.1329* | 0.0926* | 0.0594* | -0.0083 | -0.0233* | 0.0292* | -0.0279* | -0.0067 | -0.0477* | -0.0462* | 0.1713* | 1 |

Note: * denotes significance at the 5% level. See Table S3 for variables definition.
