# Supplementary material for: Female top managers and firm performance
Source: PLoS One. 2023 Feb 15;18(2):e0273976. doi: 10.1371/journal.pone.0273976 (PMC9931137; doi:10.1371/journal.pone.0273976)
Supplement: S6 Table — Results for value added per worker. (DOCX) [file pone.0273976.s006.docx]

**S6 Table. Gender bias and firm performance. Results for value added per worker**

|  | (1) | (2) | (3) | (4) |
| --- | --- | --- | --- | --- |
| **Dep. Variable:** | **Labour Productivity** | | |  |
| **Ind. Variables** |  |  |  |  |
| Female Presence | -0.068*** | -0.057*** | -0.007 | -0.007 |
|  | (0.018) | (0.019) | (0.021) | (0.021) |
| Female Top Manager |  | -0.053* | 0.178*** | 0.177*** |
|  |  | (0.028) | (0.054) | (0.055) |
| Female Presence*Top Manager |  |  | -0.342*** | -0.337*** |
|  |  |  | (0.060) | (0.060) |
| Experience of the manager |  |  |  | -0.001 |
|  |  |  |  | (0.001) |
| Ln number of workers | 0.063*** | 0.063*** | 0.060*** | 0.061*** |
|  | (0.010) | (0.010) | (0.010) | (0.010) |
| Crime | 0.000 | -0.001 | -0.001 | -0.000 |
|  | (0.008) | (0.008) | (0.008) | (0.008) |
| Informal competition | -0.017** | -0.016** | -0.017** | -0.016** |
|  | (0.007) | (0.007) | (0.007) | (0.007) |
| Corruption | 0.015** | 0.015** | 0.015** | 0.014** |
|  | (0.006) | (0.006) | (0.006) | (0.006) |
| Access to finance | -0.063*** | -0.063*** | -0.064*** | -0.064*** |
|  | (0.007) | (0.007) | (0.007) | (0.007) |
| Ln age | 0.065*** | 0.066*** | 0.065*** | 0.067*** |
|  | (0.012) | (0.012) | (0.012) | (0.013) |
| Ownership concentration | -0.307*** | -0.289*** | -0.281*** | -0.281*** |
|  | (0.032) | (0.033) | (0.033) | (0.033) |
| Exporter | 0.304*** | 0.307*** | 0.306*** | 0.303*** |
|  | (0.024) | (0.024) | (0.024) | (0.024) |
| Foreign-owned | 0.453*** | 0.454*** | 0.450*** | 0.433*** |
|  | (0.041) | (0.042) | (0.043) | (0.042) |
|  |  |  |  |  |
| Observations | 34,914 | 34,133 | 34,133 | 33,668 |
| Adjusted R-squared | 0.777 | 0.776 | 0.777 | 0.777 |
| Note: Robust standard errors in parentheses cluster by survey weights. *** p<0.01, ** p<0.05, * p<0.1. Country, sector, and year dummies are added in all models, not reported to save space. Labour productivity is defined as value added per worker. | | | | |
