# Supplementary material for: Female top managers and firm performance
Source: PLoS One. 2023 Feb 15;18(2):e0273976. doi: 10.1371/journal.pone.0273976 (PMC9931137; doi:10.1371/journal.pone.0273976)
Supplement: S7 Table — (DOCX) [file pone.0273976.s007.docx]

**S7 Table. Reduction Bias before and after matching**

|  | **Unmatched** | **Mean** | | **bias** | | **T-test** | |
| --- | --- | --- | --- | --- | --- | --- | --- |
| **Variable** | **Matched** | **Treated** | **Control** | **%** | **% reduction** | **t** | **p>t** |
| Ln number of workers | Unmatched | 3.1218 | 3.3955 | -19.9 |  | -18.2 | 0 |
|  | Matched | 3.1219 | 3.1506 | -2.1 | 89.5 | -1.5 | 0.135 |
| Ln age | Unmatched | 2.5496 | 2.6206 | -9 |  | -8.14 | 0 |
|  | Matched | 2.5497 | 2.5489 | 0.1 | 98.8 | 0.07 | 0.941 |
| Ownership  concentration | Unmatched | 0.79294 | 0.78527 | 2.9 |  | 2.65 | 0.008 |
|  | Matched | 0.79292 | 0.79612 | -1.2 | 58.3 | -0.85 | 0.394 |
| Exporter | Unmatched | 0.21611 | 0.23272 | -4 |  | -3.62 | 0 |
|  | Matched | 0.21603 | 0.22085 | -1.2 | 71 | -0.82 | 0.41 |
| Foreign-owned | Unmatched | 0.05989 | 0.07324 | -5.8 |  | -5.19 | 0 |
|  | Matched | 0.05989 | 0.06044 | -0.2 | 95.9 | -0.18 | 0.859 |
| Crime | Unmatched | 1.1266 | 1.1264 | 0 |  | 0.02 | 0.986 |
|  | Matched | 1.1264 | 1.0991 | 0.00001 | 17.18 | 1.52 | 0.129 |
| Informal competition | Unmatched | 1.4451 | 1.4667 | -1.6 |  | -1.46 | 0.144 |
|  | Matched | 1.445 | 1.4339 | 0.8 | 48.4 | 0.58 | 0.565 |
| Corruption | Unmatched | 1.5198 | 1.7605 | -16.5 |  | -15.17 | 0 |
|  | Matched | 1.5196 | 1.5139 | 0.4 | 97.6 | 0.28 | 0.779 |
| Access to finance | Unmatched | 1.4306 | 1.5037 | -5.5 |  | -5.1 | 0 |
|  | Matched | 1.4308 | 1.4267 | 0.3 | 94.5 | 0.22 | 0.829 |
| Experience | Unmatched | 15.772 | 17.284 | -14.4 |  | -13.01 | 0 |
|  | Matched | 15.773 | 15.53 | 2.3 | 84 | 1.7 | 0.088 |
