# Supplementary material for: Female top managers and firm performance
Source: PLoS One. 2023 Feb 15;18(2):e0273976. doi: 10.1371/journal.pone.0273976 (PMC9931137; doi:10.1371/journal.pone.0273976)
Supplement: S8 Table — (DOCX) [file pone.0273976.s008.docx]

**S8 Table. Additional Robustness Checks**

| **Dep variable: Labor productivity** | **(1)** | **(2)** | **(3)** | **(4)** | **(5)** |
| --- | --- | --- | --- | --- | --- |
| **Specification including:** | **Fem_Edu_Years** | **Fem_Lab_Force** | **Small** | **Medium** | **Large** |
| **Ind. Variables** |  |  |  |  |  |
| Female Presence | -0.022 | -0.032 | -0.072* | -0.029 | 0.086 |
|  | (0.042) | (0.026) | (0.038) | (0.040) | (0.064) |
| Female Top Manager | 0.252*** | 0.171*** | 0.107 | 0.224*** | 0.346** |
|  | (0.072) | (0.048) | (0.068) | (0.083) | (0.136) |
| Female Presence*Top Manager | -0.421*** | -0.337*** | -0.259*** | -0.379*** | -0.242 |
|  | (0.086) | (0.057) | (0.078) | (0.098) | (0.156) |
| Education Females | 0.040*** |  |  |  |  |
|  | (0.005) |  |  |  |  |
| Ln Female Employment |  | 0.029* | 0.016 | 0.047** | 0.082*** |
|  |  | (0.015) | (0.024) | (0.023) | (0.032) |
| Ln number of workers | -0.016 | -0.021 | 0.033 | -0.011 | -0.193*** |
|  | (0.018) | (0.017) | (0.034) | (0.045) | (0.048) |
| Crime | -0.008 | -0.014 | -0.005 | -0.024 | -0.000 |
|  | (0.017) | (0.009) | (0.013) | (0.016) | (0.025) |
| Informal competition | -0.005 | -0.040*** | -0.033*** | -0.041*** | -0.052** |
|  | (0.013) | (0.008) | (0.011) | (0.013) | (0.021) |
| Corruption | 0.016 | 0.023*** | 0.045*** | 0.010 | -0.038* |
|  | (0.013) | (0.008) | (0.011) | (0.015) | (0.021) |
| Access to finance | -0.059*** | -0.041*** | -0.020* | -0.071*** | -0.031 |
|  | (0.015) | (0.009) | (0.012) | (0.014) | (0.022) |
| Ln age | 0.076*** | 0.063*** | 0.039* | 0.076** | 0.130*** |
|  | (0.025) | (0.018) | (0.024) | (0.030) | (0.038) |
| Ownership concentration | -0.514*** | -0.317*** | -0.371*** | -0.273*** | -0.154* |
|  | (0.060) | (0.041) | (0.062) | (0.069) | (0.091) |
| Experience of the manager | -0.001 | 0.004*** | 0.002 | 0.003 | 0.008*** |
|  | (0.002) | (0.001) | (0.002) | (0.002) | (0.002) |
| Exporter | 0.353*** | 0.151*** | 0.144*** | 0.164*** | 0.150** |
|  | (0.044) | (0.036) | (0.049) | (0.055) | (0.061) |
| Foreign-owned | 0.295*** | 0.545*** | 0.418*** | 0.686*** | 0.473*** |
|  | (0.077) | (0.048) | (0.075) | (0.076) | (0.089) |
|  |  |  |  |  |  |
| Observations | 11,961 | 22,637 | 11,534 | 7,691 | 3,412 |
| Adjusted R-squared | 0.770 | 0.773 | 0.784 | 0.763 | 0.783 |

Note: Robust standard errors in parentheses cluster by survey weights. *** p<0.01, ** p<0.05, * p<0.1. Country, sector, and year dummies are added in all models, not reported to save space.
