# Supplementary material for: Female top managers and firm performance
Source: PLoS One. 2023 Feb 15;18(2):e0273976. doi: 10.1371/journal.pone.0273976 (PMC9931137; doi:10.1371/journal.pone.0273976)
Supplement: Attachment — Submitted filename: Female Top R2_PONE_ReviewCommentsAnswers.docx [file pone.0273976.s010.docx]

**Response to Review Comments to the Author**

**Reviewer #1:** The revised paper is substantially improved. However, I still have a few minor comments, mostly related to inconsistencies between the text and tables, which the authors should review and (if necessary) correct.
P. 9: ”a number similar to the average in high-income OECD and non-OECD countries, slightly lower in SSA (28 percent).” -> According to Table 1, the SSA estimate is 29 percent. **Thanks, corrected.**
P. 9: “Finally, the third gender variable, female dominated ownership, is shown in columns 3 and 6 for world regions.” -> I don’t see a column 6 in Table 1.

**Thanks, I have deleted “and 6”.**
P. 10: “In terms of total sales, a significantly higher number of firms are managed by males in most regions, except SAR.” -> I don’t understand this sentence. My reading of Table 3 is that male-managed firms have higher sales than female-managed firms in most regions, except SAR.

**The sentence has been modified accordingly.**
P. 10: “In Table 4, we show the participation of women in firms classified by employment size. It can be observed that a woman’s presence in ownership is more common in small and medium firms in both developed and developing countries, whereas in the MENA region, the reverse is the case.” -> My reading of Table 4 is that “female presence in ownership is *not* more common in small and medium firms in developing countries” (i.e., small = 29.8%; medium=32%; large=36%). Also, the text should be rephrased to make it clear that MENA results are not actually shown in the Table.

**Many thanks for the suggested correction, it has been taken on board and the MENA part has been deleted.**
P. 14: The paper argues that “if females are among the owners and the top manager is a female, the average labor productivity is only around 1.2 percent lower (0.098-0.110=-0.012) according to column (3).” -> it would be useful to test if the combined effect (i.e., 0.012) is statistically different from zero.

**Thanks for this suggestion: A different regression has been estimated to directly obtain the coefficient of the combined effect and the corresponding standard error, and the coefficient is significant at the 5% level. A footnote (7) has been added in the revised manuscript.**
P. 15: “The first interesting outcome is that female presence in ownership when the top manager is a male shows a positive and significant effect on labor productivity in SSA, MENA, and SAR regions and a negative effect on ECA.” -> There is also a negative effect in EAP (marginally significant).

**I have added this fact also in the text, thanks.**
P. 21: How is labor productivity defined in Tables 2 and 3? Is this sales per workers? If yes, then it should be labeled as such since value added per worker is also a measure of labor productivity (and is described as such in the text, e.g. p. 10: “In terms of value-added per worker (labor productivity), no significant differences are found for the EAC, SSA, and EAP regions, whereas for LAC and MENA, the performance is higher for male-managed firms.”). Tables 2 and 3 should use the same variable names.

**Both changes have been made (labor productivity has been replaced by sales per worker and the variables in Table 3 are now taken from Table 2, thanks.**
